# VMP1-deficient Chlamydomonas exhibits severely aberrant cell morphology and disrupted cytokinesis

**DOI:** 10.1186/1471-2229-14-121

**Published:** 2014-05-06

**Authors:** Hezi Tenenboim, Julia Smirnova, Lothar Willmitzer, Martin Steup, Yariv Brotman

**Affiliations:** 1 Institute of Biochemistry and Biology, Department of Plant Physiology, Universität Potsdam, Potsdam, Germany; 2 Max Planck Institute for Molecular Plant Physiology, Am Mühlenberg 1, 14476 Potsdam, Germany

**Keywords:** VMP1, Autophagy, Cytokinesis

## Abstract

**Background:**

The versatile Vacuole Membrane Protein 1 (VMP1) has been previously investigated in six species. It has been shown to be essential in macroautophagy, where it takes part in autophagy initiation. In addition, VMP1 has been implicated in organellar biogenesis; endo-, exo- and phagocytosis, and protein secretion; apoptosis; and cell adhesion. These roles underly its proven involvement in pancreatitis, diabetes and cancer in humans.

**Results:**

In this study we analyzed a VMP1 homologue from the green alga *Chlamydomonas reinhardtii*. CrVMP1 knockdown lines showed severe phenotypes, mainly affecting cell division as well as the morphology of cells and organelles. We also provide several pieces of evidence for its involvement in macroautophagy.

**Conclusion:**

Our study adds a novel role to VMP1's repertoire, namely the regulation of cytokinesis. Though the directness of the observed effects and the mechanisms underlying them remain to be defined, the protein's involvement in macroautophagy in *Chlamydomonas*, as found by us, suggests that CrVMP1 shares molecular characteristics with its animal and protist counterparts.

## Background

VMP1 (Vacuole Membrane Protein 1) is a transmembrane protein with homologues in all eukaryotic kingdoms, with fungi being a notable exception. It has been shown to be located in several cellular compartments: the Golgi apparatus [1], the endoplasmic reticulum [1,2], autophagosomes [3], and the plasma membrane [4]. It has been implicated in an array of cellular processes: organellar biogenesis, endo-, exo-, phagocytosis and protein secretion [2]; apoptosis [1]; adhesion [4]; and, perhaps most notably, macroautophagy (henceforth: "autophagy"; [3]), as well as a special type of autophagy, namely zymophagy, in which zymogen granules—subcellular structures that contain inactive digestive enzyme—are selectively degraded [5]. Last but not least, it has been shown to play a key role in certain human diseases, namely pancreatitis [1,6], diabetes [7], and several types of cancer [4,8,9]. The phenotypes and processes in which VMP1 is involved are gradually being elucidated at the molecular level: its plasma-membrane localization and cell-adhesion capacity, for instance, account for its reduced expression in metastasized cancer cells [4], since loss of adhesion is essensial for metastasis [10]; and its role in zymophagy helps to protect pancreas cells from digesting themselves during pancreatitis [5].

In this work we identified a VMP1 homologue (henceforth named CrVMP1) in the green alga *Chlamydomonas reinhardtii*. Partial silencing of the *CrVMP1* gene resulted in severe phenotypes, mainly affecting cell division and morphology. VMP1 mutants in other organisms have thus far never shown any detrimental effects concerning these two cellular aspects, raising curious questions regarding the evolution of the protein's role across the various eukaryotic kingdoms.

## Results

### CrVMP1

**Figure 1:**
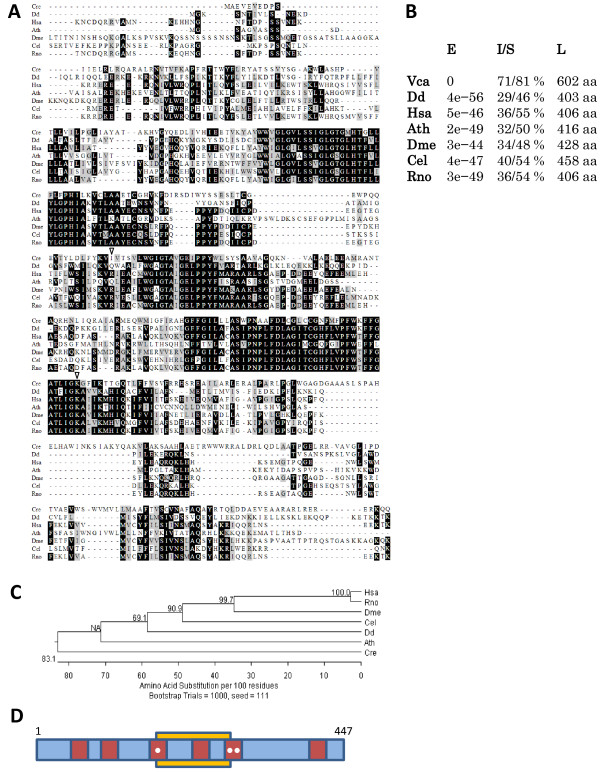
**Sequence analysis of CrVMP1. (A)** Sequence alignment (ClustalW) of CrVMP1 and its six reported homologues from *Dictyostelium discoideum* (Dd), *Homo sapiens* (Hsa), *Arabidopsis thaliana* (Ath), *Drosophila melanogaster* (Dme), *Caenorhabditis elegans* (Cel), and *Rattus norvegicus* (Rno). Black shading denotes identical residues, grey shading—similar residues. Most of the homologue residues aligned before CrVMP1's first residue were omitted. Empty arrowheads point to the first and last residues of CrVMP1's SNARE domain. **(B)** A list of CrVMP1's six reported homologues, as well as its closest homologue (in *Volvox carteri*), along with their homology's Expect value (E), the percentage of identical/similar amino acids (I/S), and their length (L). **(C)** Phylogenetic tree of the seven reported VMP1 homologues. Sequences were first aligned with ClustalW, the tree then prepared with DNASTAR MegAlign using a bootstrap test with 1,000 iterations (bootstrap percentage values are indicated at each node. NA (not applicable) appears because MegAlign always generates rooted phylogenetic trees, whereas the Clustal algorithms produce un-rooted trees. In order for MegAlign to display the Clustal tree, it must first root the tree, which involves introducing a new node. When the tree is then re-rooted, if the new node lands on a branch within the rooted tree, it does not have a bootstrapping value because it was not included in the analysis). **(D)** Schematic representation of CrVMP's primary structure. Red areas represent transmembrane domains predicted by TMPred and TMHMM. One circle represents a domain absent from the Phobius prediction, two circles—absent from both the Phobius and the MINNOU predictions. The yellow area shows the predicted SNARE domain.

*CrVMP1* (Cre06.g272000) is the only member of its family in the *Chlamydomonas reinhardtii* genome—similarly to most organisms investigated in regard to *VMP1*, with the two *KMS* genes of *Arabidopsis thaliana* being a notable exception [11]. The protein, whose coding gene is located on chromosome 6, is similar in length, sequence and structure to its six reported homologues (Figure 1A,B). CrVMP1 is predicted to harbor between four and six transmembrane domains, as predicted by TMHMM [12], TMPred [13], MINNOU [14] and Phobius [15], as well as two coiled-coil domains, as predicted by COILS [16], the latter often indicating protein-protein interaction. In addition, a 111-aa SNARE-associated Golgi domain is predicted in the protein's center (Figure 1D). As for prediction of subcellular localization, six different algorithms produced highly ambiguous and at times contradictory results: nearly equal chances were given to almost every possible cellular compartment as candidate for CrVMP1's localization—the ER, Golgi apparatus, plasma membrane, thylakoid membranes, and various vacuoles (data not shown). CrVMP1 sports an RKXX motif at its C-terminus, which makes it a strong candidate for ER localization [17].

### VMP1 knockdown cells show severe phenotypes

We used artificial miRNA [18,19] in an attempt to silence *CrVMP1*. Two *C. reinhardtii* strains were used for silencing: CC-4350, better known as cw15 302, a cell-wall-deficient strain that shows high transformation efficiencies; and UVM11, a derivative of CC-4350 that had undergone UV mutagenesis with the purpose of enhancing its capacity for expressing foreign genes [20]. We analyzed *CrVMP1* mRNA levels in our lines using qRT-PCR. In transformed clones mRNA levels varied greatly; all the clones we used in our experiments exhibited *CrVMP1* mRNA levels that ranged between 5–25% of WT and of empty-vector control.

**Figure 2:**
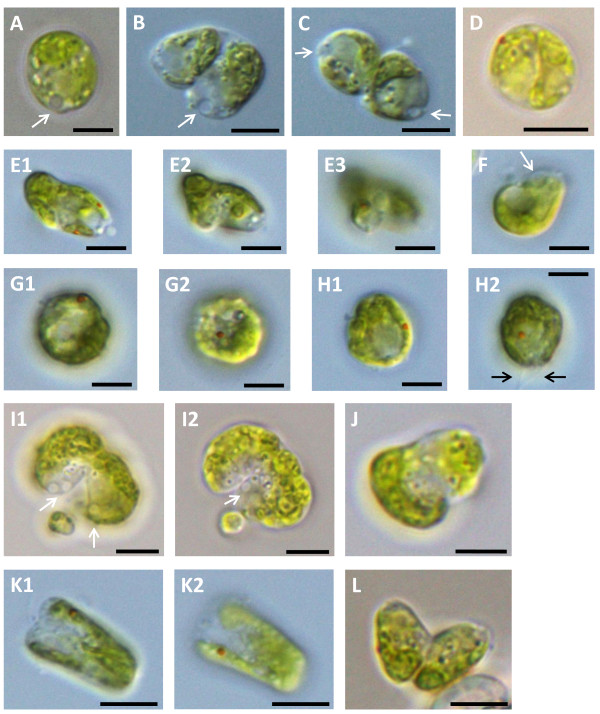
**VMP1-deficient CC-4350 cells display severe phenotypes. (A)** WT cell. **(B − L)** VMP1-deficient cells. Images labeled with the same letter show the same cell, using different focal planes, done in order to reveal more details. White arrows point at CVs; black arrows point at flagella. Scale bars = 5 μm.

**Figure 3:**
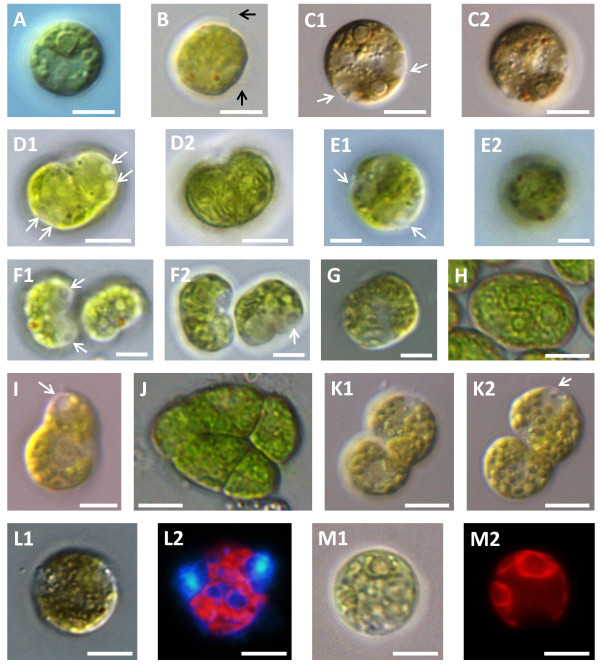
**VMP1-deficient UVM11 cells display severe phenotypes. (A)** WT cell. **(B − M)** VMP1-deficient cells. (L2, M2) Epifluorescence images of L1 and M1, respectively. Blue areas in L2 display DAPI-stained DNA. Red areas in L2 and M2 show chlorophyll autofluorescence. Images labeled with the same letter show the same cell, using different focal planes, done in order to reveal more details. White arrows point at CVs; black arrows point at flagella. Scale bars = 5 μm.

We first subjected our mutants to microscopic analysis. All knockdown lines displayed an array of striking, severe phenotypes (Figures 2 and 3). The hallmark of the mutant phenotype was defective cytokinesis. In these cells division was progressing in an abnormal fashion, resulting in daughter cells that were aberrantly attached to each other, often with visible division furrows (Figure 2B,C,I,L; Figure 3D,F,J,K).

Additionally, and often concomitantly, cells exhibited aberrant organelle numbers. The organelles in question were pyrenoids (Figure 3C,H) and eyespots (Figure 2E,G,H,K; Figure 3B,C,E,G), of which many of our knockdown cells had two or more (compared with one pyrenoid and one eyespot in WT cells), contractile vacuoles (Figures 2C,I; Figure 3C,D,E,F; two or more pairs in knockdowns, one pair in WT), and nuclei (Figure 3L; two in knockdowns, one in WT). The latter were detected by staining the cells with the fluorescent DNA dye 4',6-diamidino-2-phenylindole (DAPI), followed by epifluorescence microscopy (Figure 3L). The binucleated cells could hardly be mistaken for WT cells undergoing normal mitosis: the nuclei were abnormally positioned, the cells were perfectly round and devoid of cleavage furrows, and lastly, the images were taken at a time point in which almost no cell underwent mitosis, indicating that the binucleated cells were a several-hour-old remnant from the previous round of division.

Multiple pyrenoids per cell were frequently easily discernible in brightfield microscopy. In epifluorescence microscopy, however, many cells that looked normal in brightfield and whose pyrenoid number was hard to determine visually would often reveal, by means of the characteristic cavity in the center of the red-autofluorescing chloroplast, that they indeed harbored two pyrenoids (Figure 3M). The aberrant numbers of nuclei, pyrenoids, eyespots and CVs again suggest defective cytokinesis.

Many of our mutant cells displayed aberrant cell shapes and internal structures. Cells of the former category failed to maintain the usual round or oval external shape of WT *C. reinhardtii*, but rather appeared in an impressive variety of irregular shapes (Figure 2E,F,I,J,K). In cells with aberrant internal features, the usual structures and organelles that are the trademark of WT *C. reinhardtii*, especially the cup-shaped chloroplast, appeared deformed and misshapen (Figures 2 and 3). We postulate that defective cytokinesis again was the culprit, as organelles misdeveloped and undetached daughter cells pressed against each other to cause deformities. This, however, bears further investigation.

Lastly, two additional phenotypes appeared on occasion: a small proportion of the cells were highly vacuolated, presumably an indication of undergoing cell death (Figure 2B). More rarely, cells were brimming with numerous globules, deeming any organelle unrecognizable (Figure 3K). This latter phenotype appeared also in WT cells, though much more rarely than in the mutant.

All the mentioned phenotypes appeared both in strain CC-4350 and UVM11, with a tendency of the former toward gross deformities and the latter toward milder, WT-like cell forms with double pyrenoids and nuclei. No correlation was observed between residual mRNA levels, as mediated by qRT-PCR, and phenotypic strength. Penetrance never reached 100%; great variation in the ratio of cells showing aberrant phenotypes was observed. We found no correlation between growth conditions (temperature, light intensity and regime, shaking speed, carbon source, culture age) and penetrance. Although CrVMP1 may be potentially involved in the cell cycle and thus be differently expressed in different stages of the cycle, we saw no difference in phenotypic strength or in *CrVMP1* mRNA levels when assayed periodically in an attempt to capture representative stages (data not shown). The milder phenotypes, such as double pyrenoids and nuclei, appeared at times in up to 90% of the cells, whereas extreme deformities showed a penetrance of around 20% (Additional file 1: Table S1). We hypothesize that the lower penetrances were a combined result of low visibility of some of the phenotypes, the influence of the cell-cycle stage of individual cells, as well as stochastic effects.

The cytokinetic defects notwithstanding, our mutants were not notably retarded in growth. Mutant liquid-cultures grew slightly, but not significantly, more slowly, and after a while always reached WT cell-densities (data not shown), indicating that most mutant cells eventually underwent successful cytokinesis. In addition, our mutants exhibited no defects in the CV or in osmoregulation, as reported in VMP1-deficient slime mold [2]. To test this, cells were immersed in pure water and incubated for several hours, with WT and mutant showing similar survival rates (data not shown). Incidentally, the clearly visible CVs in our images often helped us orient inside the more grossly deformed cells, in which organelles and cell polarity were hardly recognizable (for instance, Figure 2B,F,I; Figure 3D,E,I).

### Electron microscopy reveals internal cellular defects

**Figure 4:**
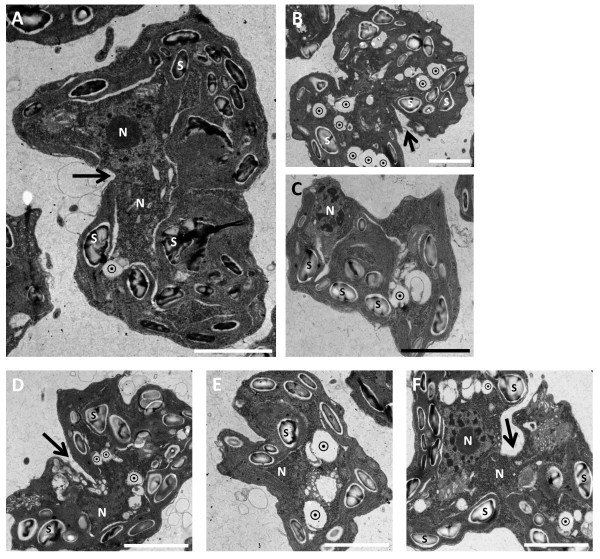
**Transmission electron microscope analysis of VMP1-deficient cells.** All panels show abnormally shaped knockdown cells (for a WT cell, see Figure 5A) at various stages of cytokinesis, with visible division furrows (black arrows). Note the mostly clear vacuoles (⨀), the often numerous and abnormally large starch granules (S; representative specimens), and the oddly placed and often deformed nuclei (N). Scale bars = 2 μm.

**Figure 5:**
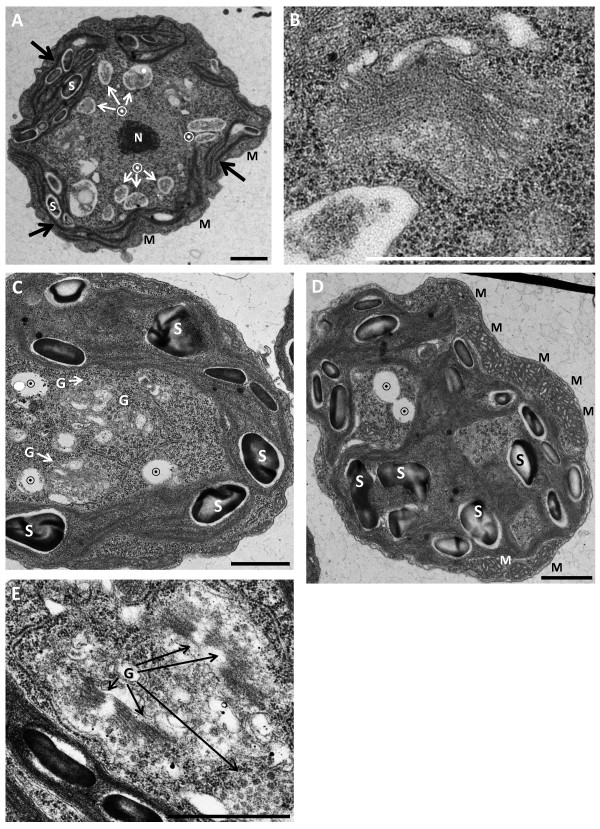
**Transmission electron microscope analysis of WT (UVM11) and VMP1-deficient cells. (A)** a WT cell. Note the electron-dense vacuoles, presumably autolysosomes (⨀), the modestly sized starch granules and mitochondria (S and M, respectively; representative specimens), the well-defined and properly located nucleus (N), and the stacked thylakoid membranes (black arrows). **(B)** A Golgi apparatus from a WT cell. **(C–D)** Two mutant cells. Note the mostly clear vacuoles (⨀), the often numerous and abnormally large starch granules, the enlarged mitochondria, and the Golgi apparatuses (G), at times only visible through their transport vesicles. **(E)** Close-up of Golgi apparatuses from a third mutant cell. Scale bars = 1 μm.

We next subjected our mutants (only in strain UVM11) to transmission electron microscopy (TEM) analysis. The resulting images revealed numerous irregularities, and concurred with our light-microscopy observations: many cells displayed defective cytokinesis and grotesque cell morphologies (Figure 4). Many internal defects, mostly inevident in light microscopy, came to light in TEM. Mutant cells often had deformed and misplaced nuclei (Figure 4). Golgi apparatuses were abnormally numerous: 3–4 per cell (Figure 5C,E), compared to almost always one in WT (although many *C. reinhardtii* strains often exhibit two apparatuses, our UVM11 cells seldom did). In many cases the Golgi seemed deformed (Figure 5C, lowest apparatus). In the mutant mitochondria were excessively large, sporting areas up to threefold greater than WT mitochondria, and featuring markedly dilated cristae (Figure 5D). Many vacuoles were visible in both WT and mutant; in the mutant they were almost always vacant (Figures 4 and 5), in WT by contrast they often contained electron-dense matter, likely membranous and cytoplasmic particles (Figure 5A). These matter-containing vacuoles are most likely autolysosomes, and their absence in the mutant may indicate a defect in autophagy. The chloroplast in the mutant appeared disorganized and misshapen, with numerous protrusions (Figure 4); the stacked thylakoid membranes (always visible in WT as dark lines within the chloroplast; Figure 5A), were often absent or much weaker in the mutant. Lastly, mutants seemed to accumulate more starch granules than WT, with some granules being excessively large (Figures 4 and 5). To test whether starch content indeed differed between WT and mutant, we performed enzymatic starch quantification, both in the end of a 12-hour light period, when starch levels peak, and in the end of the dark peroid, when most starch is degraded. We observed no significant difference between WT and mutant with regard to starch content (Additional file 2: Figure S1).

### Several cell-cycle and autophagy regulators are underexpressed in VMP1 mutants

**Figure 6:**
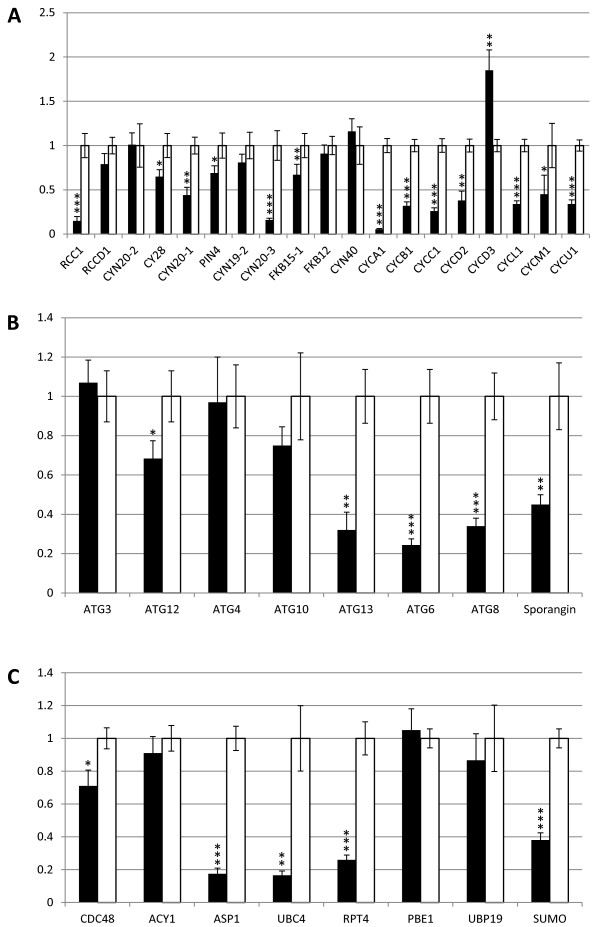
**Expression levels of selected cell-cycle, autophagy and protein-degradation genes.** Values are shown as fold expression. Expression levels in empty-vector control (white columns) is always 1; black columns represent expression levels in the VMP1-deficient mutant. **(A)** Expression of selected regulators of the cell cycle. **(B)** Expression of autophagy regulators and markers and of sporangin. **(C)** Expression of genes involved in protein degradation. Expression levels were measured by qRT-PCR. Error bars represent standard deviations from three technical replicates. Stars represent the statistical significance for each gene: (no stars) 0.05 < *p*, (*) 0.01 < *p* < 0.05, (**) 0.001 < *p* < 0.01, (***) *p* < 0.001.

Given that the microscopic phenotypes pointed toward defective cell division, we sought to assay for the expression in the mutant of several key regulators of the cell cycle. In addition, VMP1's strong involvement in apoptosis [1] and autophagy in humans [3] and slime mold [21] led us to assay for the expression of several autophagy markers, as well as genes participating in protein degradation. Transcript levels were heterogeneous, with several genes retaining WT-like expression levels, and others exhibiting significant, at times extreme, downregulation in the knockdown (Figure 6). Knockdown mRNA levels of three cell-cycle regulators were less than 15% of WT: RCC1 (Regulator of Chromosome Condensation), CYN20-3 (of the cyclophilin family), and CYCA1 (cyclin-dependent protein kinase regulator) (Figure 6A). Two genes whose products participate in protein degradation, the AMP-forming ubiquitin ligase *UBC4* and the aspartyl protease *ASP1*, were expressed in the mutant at less than 20% of WT (Figure 6C). Notably, two crucial autophagy-related genes were downregulated in the mutant: *ATG8* (called *LC3* in mammals), whose product is an established marker of autophagy in *C. reinhardtii*[22], and *ATG6*, whose human homologue encodes a protein, beclin-1, that interacts with human VMP1 [3] (Figure 6B). Lastly, we assayed for the expression of sporangin, the vegetative cell-wall lytic enzyme that facilitates daughter-cell hatching following cytokinesis [23]. The sporangin gene was consistently expressed in the knockdown at 40–50% of WT (Figure 6B). Taken together, these results indicate that reduced VMP1 levels result in underexpression of several key regulators of the cell cycle and of autophagy, which in turn results in defective cytokinesis and, tentatively, reduced ability to undergo autophagy.

### Metabolomic profiling reveals additional changes in mutant

**Figure 7:**
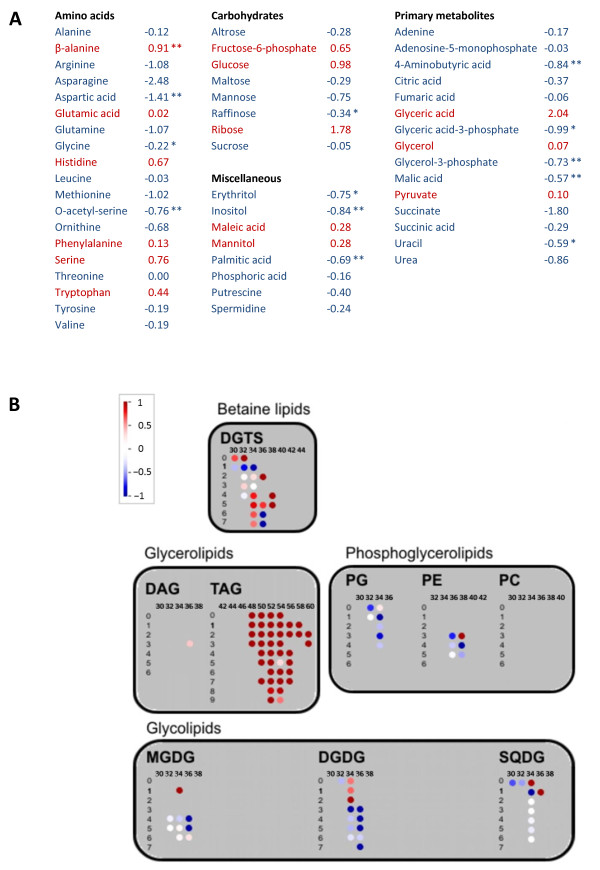
**Metabolomic profiling of VMP1-deficient cells versus empty-vector control. (A)** List of metabolites analyzed by GC-MS. The numbers represent log_2_ fold-change in the mutant compared to empty-vector control; positive numbers (red) denote accumulation in the mutant, negative numbers (blue): accumulation in empty-vector control. A single star (*) indicates *p* < 0.05; double stars (**) indicate *p* < 0.01. **(B)** Heat map of the lipids measured, sorted according to lipid class, carbon-chain length and number of double bonds. The schematic, produced by MapMan [24], shows the lipids' log_2_ fold-change in the mutant compared to empty-vector control: red circles denote lipid accumulation in the mutant, blue circles denote accumulation in empty-vector control, white circles denote nearly no change. For a comprehensive list with numeric values and statistical significance, see Additional file 4: Table S2.

To obtain a more comprehensive picture of the changes that occur in our mutant, we subjected our cells to quantitative metabolomic profiling. Metabolites were measured using both gas chromatography–mass spectrometry (GC-MS) and ultra-performance liquid chromatography-mass spectrometry (UPLC-MS). All analyses showed significant and coherent differences between WT/empty-vector control and mutant, as ajudged by principal component analysis (Additional file 3: Figure S2). All detected metabolites were used for PCA preparation, while only securely annotated metabolites are shown in the following results). GC-MS analysis resulted in thirteen metabolites showing significant changes in knockdown versus empty-vector control (Figure 7A), whereas UPLC-MS analysis resulted in numerous lipids showing changed levels (Figure 7B; for numeric values and statistical data, see Additional file 4: Table S2). Most notable was the mutant's stark accumulation of triacylglycerides (TAG), ubiquitous storage lipids. Other lipid classes were less coherent, but still meaningful: most detected diacylglyceroltrimethylhomoserine (DGTS) lipids, which replace phosphatidylcholines in *C. reinhardtii*, accumulated in knockdown cells, whereas most digalactosyldiacylglycerols (DGDG), phosphatidylethanolamine (PE) and phosphatidylglycerols (PG), all serving mostly as membrane lipids, showed higher levels in WT than in the mutant.

## Discussion

VMP1 has been researched before in a plant (*A. thaliana*; [11]) and in a microorganism (*D. discoideum*; [2]), and now: in a species that is both. In this work we were able to assign a novel role to this already versatile protein. VMP1 has thus far not been implicated in relation to defective cell division. At the same time, a rather modest number of cytokinesis mutants in *Chlamydomonas* has been reported [25]. Some were natural isolates [26], others generated by and identified following nonspecific mutagenesis [27], while others yet were the result of targeted silencing [28-30]. In the three latter examples, proteins of the basal bodies, cytoskeleton, and flagella, respectively, were silenced. Indeed, these three structures are tightly related to cell division; processes such as flagellation and cleavage-furrow formation, formerly thought to be completely distinct, are gradually losing the borders between them, and proteins that are associated to these structures often exhibit multiple roles. Not much can be deduced though with regard to the cytoskeleton, basal bodies and flagella in our mutant. As for the latter, our background *Chlamydomonas* strains, CC-4350 and UVM11, harbor very short flagella; often no flagella were observed under the microscope, anecdotally more so in the mutant than in WT, but it was impossible for us to determine whether this should be ascribed to the VMP1 deficiency or rather to limited visibility.

Another organelle starring in our knockdown cells was the eyespot (Figures 2 and 3). It has been reported that a few cells with two eyespots can frequently be seen in WT cultures. True mutants that harbor multiple eyespots (as well as mutants that have unusually small eyespots and ones that have no eyespots at all) have also been reported [31]. Our mutant cells were markedly different: they almost always showed cell-division defects (cleavage furrows, deformed chloroplasts) in addition to harboring multiple eyespots; and they lacked the *mlt1*^*−*^ characteristic of usually having one correctly placed eyespot and an additional, aberrantly placed eyespot near the base of the flagella [31].

Electron microscopy revealed in the mutant enlarged mitochondria, disorganized chloroplasts, autolysosomes in WT but not in the mutant, overnumbered and perhaps misshapen Golgi apparatuses, and excessively large starch granules (Figures 4 and 5). The latter two phenomena are known to be linked: plants tend to accumulate starch when their Golgi system is inhibited or disrupted [32]. However, claiming that such is the case in our mutant would be unsubstantiated: our EM images do not allow for a clear-cut definition of the Golgi's well-being or lack thereof in our cells. That said, the increased number of Golgi apparatuses in our mutant (Figure 5C,E) points again toward abnormality. The same applies for starch: enzymatic quantification showed no difference in starch content between WT and mutant (Additional file 2: Figure S1), and our EM images allow no statistical analysis of the amount or size of starch granules. This aspect of our phenotype therefore remains anecdotal and bears further investigation. A connection between VMP1 and Golgi in *Chlamydomonas* would correspond to observations in the former's homologues: VMP1 was shown to be localized to the Golgi apparatus in the first VMP1 study [1], and VMP1-deficient slime mold showed morphological and functional Golgi defects [2].

VMP1 has been shown several times to be an inducer of autophagy, and the molecular mechanisms by which it so acts are gradually being elucidated [33]. It has been shown that VMP1, a multispan transmembrane protein, is anchored to the ER membrane and is essential for autophagosome formation, as siRNA-VMP1 knockdown cells hardly form any autophagosomes, even under autophagy-inducing conditions, such as starvation and rapamycin treatment [3]. Autophagy, despite being extensively researched and discussed—albeit mostly in animals—still bears many unknowns; doubly so in *Chlamydomonas*, in which the topic is in its infancy [22]. In our study we tentatively show that autophagy may be downregulated in *Chlamydomonas* VMP1 knockdown cells (it should be noted that autophagy was never actively induced in our experiments. The observed autophagic phenomena in our WT cells represent basal autophagy, possibly in combination with slight, unintentional nutrient deprivation as a function of the culture's age). With regard to the observed underexpression of autophagy markers (Figure 6B), we identified with interest VMP1's interaction partner in humans, beclin-1 (named ATG6 in yeast), as one of the genes downregulated in our knockdown. The VMP1-beclin-1 interaction in human was shown to be essential for the induction of autophagy [3]. It would be of interest to test whether a regulatory adaptation of beclin-1 levels to VMP1 levels in the cell accounts for the former's decreased levels in our mutant.

Further evidence for compromised autophagy was delivered by TEM in the form of autolysosomes that were present in WT but nearly absent in the mutant (Figures 4 and 5), and of grossly enlarged mitochondria in the mutant (Figure 5D). It has been shown that inhibited autophagy results in the accumulation of enlarged mitochondria in rat myoblast cells [34]. Mitochondria frequently undergo fission in healthy cells, but a fraction fail to do so, for unknown reasons. These larger, non-fissioned mitochondria are presumably discriminated against by the autophagic (more precisely: mitophagic) machinery, since the engulfment of large organelles by autophagosomes requires more energy than that of smaller organelles. This is exacerbated by compromised autophagy, such as occurs in scenecent cells or in experimental inhibition of autophagy: clearance of larger mitochondria by means of autophagy is reduced even more, and soon enough they become a majority [35]. The increased levels of reactive oxygen species that enlarged, damaged mitochondria produce [36] in turn damage the autophagic machinery even more.

The accumulation of starch, as seen in our TEM images, may also be a sign of disrupted autophagy: the aberrant accumulation of glycogen, starch's animal counterpart, in three genetic human diseases—Lafora disease [37], Pompe disease [38], and Danon disease [39]—with detrimental pathophysiologies, has been shown in the cited studies to be a result of compromised autophagy. More recently and relevantly, disrupted/inhibited autophagy resulted in starch accumulation in leaves of *Arabidopsis thaliana* and *Nicotiana benthamiana*[40]. Indeed, no excess of starch was found in our mutant when quantified enzymatically (Additional file 2: Figure S1); however, the accumulation of starch in excessively large granules may not necessarily correspond to an overall increase in cellular starch content. Our last piece of evidence is reflected in the results of our lipidomic profiling (Figure 7B), whereby the accumulation of TAGs in our mutant may be ascribed to defective autophagy [41], a result, according to the cited study, of reduced hydrolysis of TAGs by defective autophagosomes. The changes we observe in other classes of lipids may also be related to autophagy, especially in the context of lipids that are constituents of the autophagosome and autolysosome membranes. Out of six phosphatidylethanolamine (PE) species that we deteceted, four were reduced in the mutant (two considerably, two mildly), one was unchanged and one accumulated in the mutant. PE is the major lipid involved in the initial stages of autophagosome formation, and it conjugates with ATG8 [42]. However, conclusions to be drawn from that must remain tentative: it is not known how paucity or plethora of autophagosomes influence the overall levels of PE in the cell; it is also not known which other lipid classes are major components of autophagosomes and autolysosomes. Incidentally, PE has also been shown to be essential for cytokinesis: Chinese hamster ovary cells depleted of PE showed severe cytokinesis defects [43].

Further experiments are required in order to provide a more definite conclusion in regard to the autophagic capacity of our mutant: ATG8 is better used in its Western-blot rather than its qRT-PCR version as a marker for autophagy; additionally, numerous reasons can account for the accumulation of TAGs, indeed a common stress response in many organisms; a direct link between the accumulation that we observe and defective autophagy still requires establishment. In this regard, our results seem to contradict several studies that showed that the massive TAG and starch accumulation that occurs in nitrogen-deprived *Chlamydomonas* is accompanied by very pronounced autophagy [44]. If TAG and starch accumulation go hand in hand with autophagy, why do we observe the two former phenomena in our supposedly autophagy-compromised cells? We postulate that the effects reported in the cited, and other, studies, indeed prerequisite nitrogen starvation, a dramatic onslaught that forces the cell to degrade proteins in order to replenish its amino-acid stock, this by means of autophagy. Basal autophagy, such as in our experiments, presumably behaves differently. As mentioned above, Singh et al. [41] found that disrupted autophagy results in TAG accumulation in rat and mouse, and, conversely, that induced autophagy results in decreased TAG levels. This has been shown in *Chlamydomonas* as well: applying the autophagy inhibitor 3-methyladenine resulted in TAG accumulation in both nitrogen-deprived and control cells [45], whereupon the author concluded that TAG accumulation in nitrogen-deprived cells may not be a direct result of autophagy.

Another observation combines our microscope images and our lipidomic profiling. A small fraction of our cells displayed a highly globulized phenotype (Figure 3K). This occurred in WT as well, but much more frequently in the VMP1 knockdown. These cells greatly resemble the lipid-body accumulating cells, following nitrogen deprivation, seen in Goodson et al. [46]. Presumably nitrogen deprivation occurred in a fraction of our cultures, resulting in massive accumulation of lipid bodies, which, incidentally, contain mainly TAGs (such cultures, it should be noted, were never used in any other experiment). The fact that this occurred much more frequently in the VMP1 knockdown reinforces the finding of TAG accumulation mediated by our analytical profiling, and again contradicts, if indeed autophagy is compromised in our cells, the findings reported by Wang et al. [44].

Our results show concurrence with observations in other organisms with regard to VMP1's involvement in autophagy, but also suggest a heretofore unreported involvement in cell division. It is interesting to note that despite the relatively high sequence conservation between the different VMP1 homologues, CrVMP1 occupies a phylogenetic branch distinct from the other six reported homologues (Figure 1C). Plausibly its sequence divergence is responsible for its added roles in *Chlamydomonas*. That said, the two hallmarks of our mutant phenotype, defective cytokinesis and defective autophagy, may be tightly linked to each other. Two recent reports provide such a link. In the first, inhibition of autophagy resulted in cytokinesis failure, multinucleation and aneuploidy in human cells [47]. According to this report, the cell uses autophagy to maintain correct levels of RhoA, a small GTPase protein critical for cytokinesis. The second report shows that inhibited autophagy in yeast results, under conditions of starvation, in aneuploidy, abnormal mitosis, and growth deficiency [48]. An even more direct link between cytokinesis and VMP1's specific role in autophagy is provided by phosphatidylinositol 3-phosphate (PI3P). The synthesis of this lipid at the preautophagosomal site is essential for autophagosome formation and is mediated by the enzyme PI3-kinase class III, which forms a complex with, among others, VMP1 [49]. PI3K-III has been shown to form complexes that regulate cytokinesis, and that are very similar to the autophagy-related PI3K-III complexes. Downregulation of various components of the PI3K-III complex resulted in impaired cytokinesis [50,51]. It is therefore possible that cytokinesis in our mutant is inhibited either as a result of impaired autophagy, whereby the VMP1 deficiency is solely the cause for said impairment; or that the VMP1 deficiency causes the cytokinesis defect in a more direct fashion, possibly through the disruption of PI3K-III complexes. Thus far, VMP1's strong involvement in cancer in humans, a disease whose hallmark is cell division gone awry, has been shown to be a result of the protein's roles in cell adhesion [4] and in autophagy [52,53]. Our results may point toward a more direct role of VMP1 in cell division.

## Conclusion

In this study we generated VMP1-deficient *Chlamydomonas* and subsequently identified both a novel role for VMP1 and a new gene involved in *Chlamydomonas* cell division and autophagy. CrVMP1-deficient cells exhibited disrupted cytokinesis and aberrant morphologies. Cell-biological and biochemical evidence suggests impaired autophgay: mutant cells accumulated triacylglycerides and formed enlarged starch granules; they were devoid of autolysosomes although WT cells showed many; and they showed enlarged mitochondria. In addition, mutants underexpressed several key genes involved in cell-cycle regulation and autophagy.

## Methods

### Strains and cultivation

The cell-wall-deficient *C. reinhardtii* strains CC-4350 (cw15 302) and UVM11 [20] were used in this study. Cells were grown in Tris-acetate-phosphate (TAP) medium [54] and were cultured in 22°C, with light intensity of 85 μmol/m^2^/sec, while shaking at 125 rpm, and, unless stated otherwise, under continuous light. Growth plates were prepared with TAP and 1.5% agar.

### Generation of VMP1 knockdown lines

Gene silencing was performed by amiRNA as described [18]. Briefly, the following pair of oligonucleotides was generated by submitting the identifier 187038 to the online WMD3 tool [55] (http://wmd3.weigelworld.org/): CTAGTTTGAGGTAGAGGATCTTTCAATCTCGCTGATCGGCACCATGGGGGTGGTGGTGATCAGCGCTATTGAGGGATCCTCTACCTCAAG and CTAGCTTGAGGTAGAGGATCCCTCAATAGCGCTGATCACCACCACCCCCATGGTGCCGATCAGCGAGATTGAAAGATCCTCTACCTCAAA. The oligonucleotides were annealed to each other, then ligated into the SpeI restriction site of the pChlamiRNA2 and pChlamiRNA3int vectors. The vectors were linearized and transformed into CC-4350 and UVM11 cells, respectively, using glass beads [56]. Transformant selection was done by arginine auxotrophy for CC-4350 and paromomycin for UVM11.

### Measurement of gene expression using qRT-PCR

Total RNA was isolated using the Universal RNA Purification Kit (Roboklon, Berlin, Germany). cDNA synthesis was performed with the RevertAid First Strand cDNA Synthesis kit (Thermo Scientific, Schwerte, Germany) using 1 mg total RNA. qRT-PCR reactions were set using the SYBR Green master mix (Applied Bioscience, Darmstadt, Germany) and performed on an HT 7900 cycler (Applied Bioscience, Darmstadt, Germany). Ubiquitin and RACK1 were used, always simultaneously, as reference genes. Relative expression values were calculated using the 2^–ΔΔCt^ method [57]. Primers were designed using QuantPrime [58]. The primers used for the detection of *VMP1* mRNA are TGACGGACTGAGTTGGAAAGGC and GAGCTAGAGGCTTCTTGCGTTG. All other primers are listed in Additional file 5: Table S3.

### Microscopy

Light and fluorescence microscopy were performed on an Olympus (Hamburg, Germany) BX-51 microscope with an oil-immersion × 100 objective, using Nomarski optics. Cells were prepared for inspection as described [59]. For transmission electron microscopy, *Chlamydomonas* cells were fixed for 3 h at 4°C with 2% (v/v) glutaraldehyde in 0.1 M sodium cacodilate buffer (pH 7.4). Samples were then fixed and osmicated for 1 h in 1% (w/v) OsO_4_. They were then stained for 2 h in 2% (w/v) uranyl acetate and dehydrated progressively in 50, 75, 95, and 100% ethanol, followed by two washes in 100% propylene oxide. The samples were embedded in Spurr's low-melting epoxy resin. Samples were degassed and cured at 60°C for 24 h. Sections (50 nm) were obtained with a Leica UC 6 ultramicrotome (Leica Microsystems, Wetzlar, Germany), mounted on 200-mesh nickel grids, counterstained with uranyl acetate followed by lead citrate, and examined with an energy-filter transmission electron microscope (EFTEM) at 80 kV (Zeiss, Oberkochen, Germany).

### Enzymatic starch quantification

Starch was quantified as described [60]. Briefly: liquid *Chlamydomonas* cultures were centrifuged, frozen in liquid nitrogen, resuspended in 80% (v/v) ethanol, then ultrasonicated. Homogenates were heated to 80°C, centrifuged, then lyophilized. Pellets were washed with water, then resuspended in 0.2 N KOH and heated to 95°C for 1 h. 1 N acetic acid was added and the samples were centrifuged. Supernatants were mixed with amyloglucosidase and incubated at 55°C overnight. Glucose was then enzymatically quantified according to Stitt et al. [61].

### Metabolomic profiling

Gas chromatography and mass spectrometry (GC-MS) analysis was performed as described [62]. Briefly: six biological replicates from each strain were subjected to GC-MS analysis. Metabolite levels were determined using the TargetSearch software package [63]. The metabolites' retention indexes (+/−2 s) and spectra (similarity > 85%) were compared against compounds stored in the Golm Metabolome Database (GMD [64]). Secondary metabolites and lipids were extracted and analyzed as described [65] using Waters Acquity ultra-performance liquid chromatography (UPLC) coupled to a Thermo-Fisher Exactive Fourier-transformation mass spectrometer. Obtained raw chromatograms were processed using Xcalibur (Thermo-Fisher, Bremen, Germany) and Refiner MS (GeneData, Basel, Switzerland). For peak annotation, the locally developed GoBioSpace database was used [65]. Peak intensities were normalized to the total ion count.

## Competing interests

The authors declare that they have no competing interest.

## Authors’ contribution

HT planned and performed most experiments and wrote the manuscript. JS performed some experiments. MS, LW and YB supervised the study. All authors read and approved the final manuscript.

## Supplementary Material

Additional file 1: Table S1 Quantification of the phenotypes of VMP1-deficient *Chlamydomonas* strains. The percentage of cells exhibiting the listed phenotypes is shown for the two background strains CC-4350 and UVM11. Click here for file

Additional file 2: Figure S1 Enzymatic starch quantification. Cells were grown in a 12/12 h light/dark regime. Starch content was measured after the dark period and after the light period in WT (UVM11), empty-vector control (EVC), and in three independent VMP1-deficient strains. Error bars represent the standard deviation of six biological replicates. Click here for file

Additional file 3: Figure S2 Principal component analyses of (A) primary metabolites analyzed by GC-MS, (B) secondary metabolites analyzed by UPLC-MS, and (C) lipids analyzed by UPLC-MS. Shown are WT replicates (brown), empty-vector control (EV, black) and VMP1-deficient cells (M, red). The original number of replicates for each strain was six, however some were lost during preparation. The number of replicates displayed here is the actual number used in all subsequent data analysis (see Figure 7). Click here for file

Additional file 4: Table S2 List of lipids analyzed by UPLC-MS. The numbers represent log_2_ fold-change in the mutant compared to empty-vector control; positive numbers (red) denote accumulation in the mutant, negative numbers (blue): accumulation in empty-vector control. A single asterisk (*) indicates *p* < 0.05; double asterisks (**) indicate *p* < 0.01; triple asterisks (***) indicate p < 0.001. Click here for file

Additional file 5: Table S3 List of qRT-PCR primers used in this work. Click here for file
