# Supplementary material for: VMP1-deficient Chlamydomonas exhibits severely aberrant cell morphology and disrupted cytokinesis
Source: BMC Plant Biol. 2014 May 6;14:121. doi: 10.1186/1471-2229-14-121 (PMC4108031; doi:10.1186/1471-2229-14-121)
Supplement: Additional file 1: Table S1 — Quantification of the phenotypes of VMP1-deficient Chlamydomonas strains. The percentage of cells exhibiting the listed phenotypes is shown for the two background strains CC-4350 and UVM11. [file 1471-2229-14-121-S1.pdf]

**Phenotype**	**CC-4350**	**UVM11**
Two or more pyrenoids	30 %	90 %
Two or more eyespots	60 %	40 %
Two or more nuclei	30 %	90 %
Two or more CV pairs	50 %	50 %
Aberrant cell shape	30 %	20 %
Deformed internal features	60 %	70 %
Severe vacuolization	10 %	10 %
Severe globulization	0 %	5 %
