# Supplementary figures and images for: VMP1-deficient Chlamydomonas exhibits severely aberrant cell morphology and disrupted cytokinesis
Source: BMC Plant Biol. 2014 May 6;14:121. doi: 10.1186/1471-2229-14-121 (PMC4108031; doi:10.1186/1471-2229-14-121)

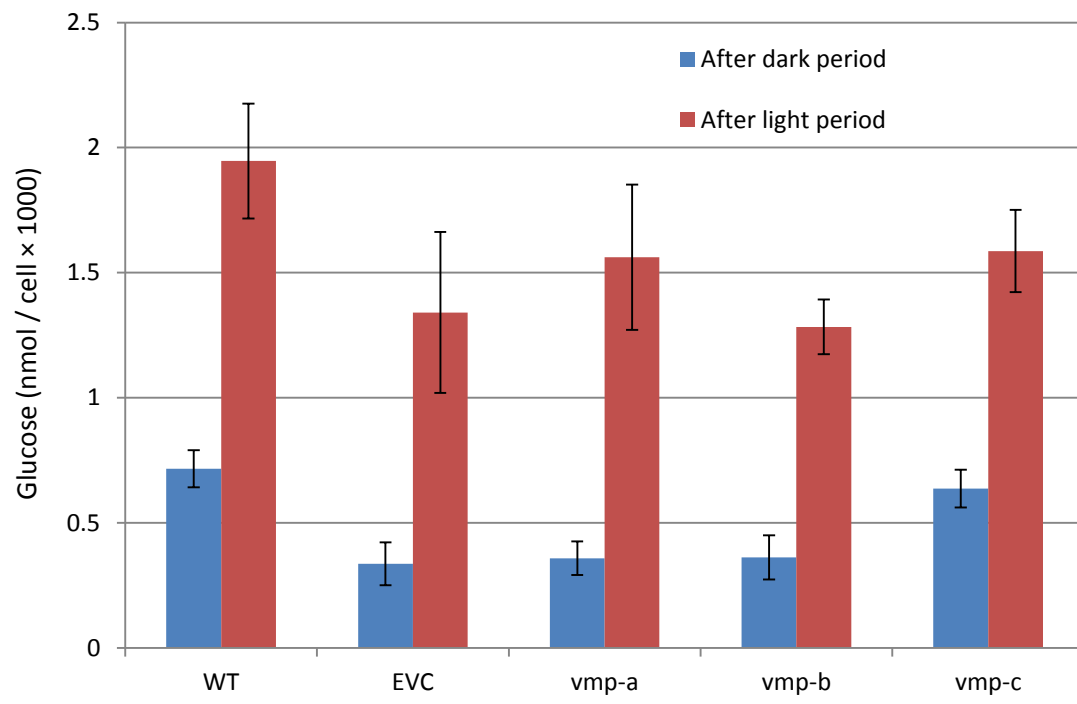

Supplement: Additional file 2: Figure S1 — Enzymatic starch quantification. Cells were grown in a 12/12 h light/dark regime. Starch content was measured after the dark period and after the light period in WT (UVM11), empty-vector control (EVC), and in three independent VMP1-deficient strains. Error bars represent the standard deviation of six biological replicates. [file 1471-2229-14-121-S2.pdf]

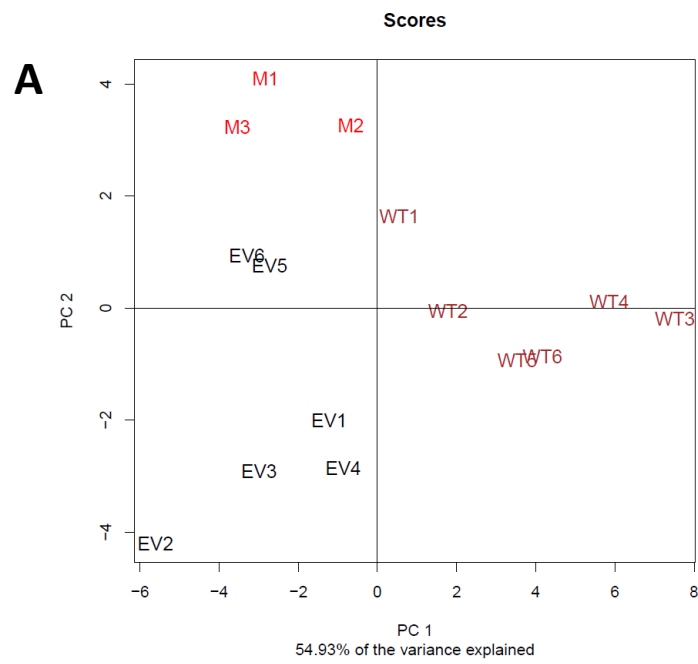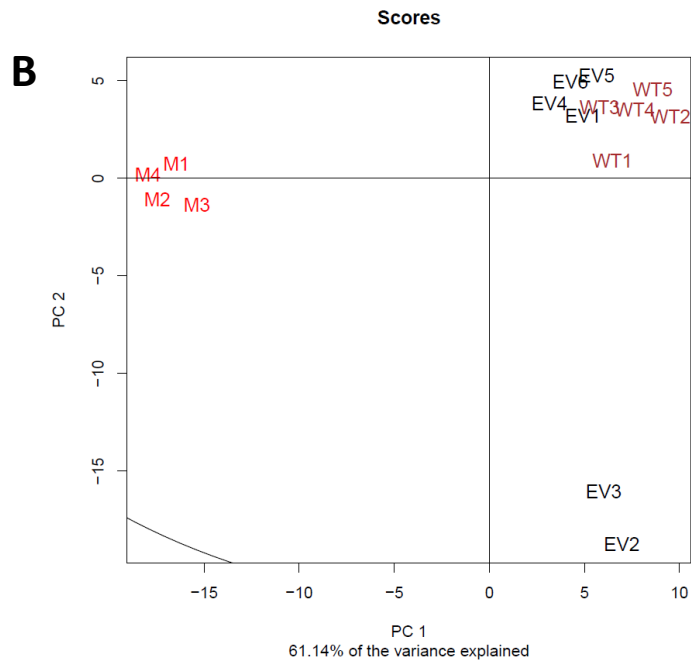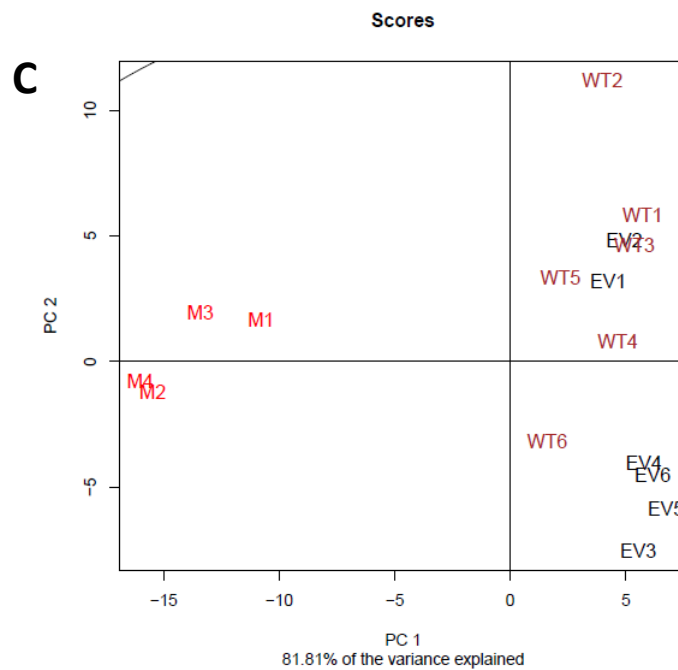

Supplement: Additional file 3: Figure S2 — Principal component analyses of (A) primary metabolites analyzed by GC-MS, (B) secondary metabolites analyzed by UPLC-MS, and (C) lipids analyzed by UPLC-MS. Shown are WT replicates (brown), empty-vector control (EV, black) and VMP1-deficient cells (M, red). The original number of replicates for each strain was six, however some were lost during preparation. The number of replicates displayed here is the actual number used in all subsequent data analysis (see Figure 7). [file 1471-2229-14-121-S3.pdf]
