# Supplementary material for: VMP1-deficient Chlamydomonas exhibits severely aberrant cell morphology and disrupted cytokinesis
Source: BMC Plant Biol. 2014 May 6;14:121. doi: 10.1186/1471-2229-14-121 (PMC4108031; doi:10.1186/1471-2229-14-121)
Supplement: Additional file 4: Table S2 — List of lipids analyzed by UPLC-MS. The numbers represent log2 fold-change in the mutant compared to empty-vector control; positive numbers (red) denote accumulation in the mutant, negative numbers (blue): accumulation in empty-vector control. A single asterisk (*) indicates p < 0.05; double asterisks (**) indicate p < 0.01; triple asterisks (***) indicate p < 0.001. [file 1471-2229-14-121-S4.pdf]

DAG 36:3	0.4
DGDG 32:0	-0.4
DGDG 34:0	0.6 ***
DGDG 34:1	0.6 *
DGDG 34:2	1.6 **
DGDG 34:3	-1.9 ***
DGDG 34:4	-0.4 ***
DGDG 34:5	-0.4 ***
DGDG 34:6	-0.3 **
DGDG 36:3	-3.2 ***
DGDG 36:4	-1.8 ***
DGDG 36:5	-1.3 ***
DGDG 36:6	-0.3 *
DGDG 36:7	-1.3 ***
DGTS 30:0	0.7 ***
DGTS 30:1	-0.4
DGTS 32:0	2.3 ***
DGTS 32:1	-0.8 ***
DGTS 32:2	0.1
DGTS 32:3	0.3 **
DGTS 32:4	-0.2
DGTS 34:1	-1.0 ***
DGTS 34:2	0.3
DGTS 34:3	0.0
DGTS 34:4	0.8 ***
DGTS 34:5	0.9 ***
DGTS 34:6	0.7 **
DGTS 34:7	0.6 **
DGTS 36:2	2.6 ***
DGTS 36:5	0.7 *
DGTS 36:6	-1.5 ***
DGTS 36:7	-1.3 ***
DGTS 38:4	2.9 ***
DGTS 38:5	4.1 ***

MGDG 32:4	-0.2
MGDG 32:5	0.1
MGDG 34:1	3.9 ***
MGDG 34:4	-0.4 *
MGDG 34:5	0.2
MGDG 34:6	0.1
MGDG 36:4	-2.1 ***
MGDG 36:5	-2.5 ***
MGDG 36:6	0.3
PE 36:3	-0.8 *
PE 36:4	-0.4
PE 36:5	-0.1
PE 38:3	1.8 ***
PE 38:4	-2.1 ***
PE 38:5	-0.5 *
PG 32:0	-0.7 *
PG 32:1	-0.1
PG 34:0	0.3
PG 34:1	-1.4 ***
PG 34:2	-0.4 **
PG 34:3	-0.9 ***
PG 34:4	-0.4 *
SQDG 30:0	-0.7 ***
SQDG 32:0	-0.5 ***
SQDG 34:0	1.0 ***
SQDG 34:1	-1.2 **
SQDG 34:2	0.0
SQDG 34:3	0.0
SQDG 34:4	-0.2
SQDG 34:5	-0.2
SQDG 34:6	0.0
SQDG 36:1	2.8 ***

TAG 48:0	1.2 **
TAG 48:1	2.0 ***
TAG 48:2	2.4 ***
TAG 48:3	2.0 ***
TAG 50:0	2.4 ***
TAG 50:1	2.2 ***
TAG 50:2	1.7 ***
TAG 50:3	1.7 ***
TAG 50:4	2.5 ***
TAG 50:5	2.8 ***
TAG 50:7	2.0 ***
TAG 52:0	3.2 ***
TAG 52:1	3.3 ***
TAG 52:2	2.6 ***
TAG 52:3	2.5 ***
TAG 52:4	1.8 ***
TAG 52:5	5.1 ***
TAG 52:6	2.1 ***
TAG 52:7	1.1 *
TAG 52:8	0.9 **
TAG 52:9	2.5 ***
TAG 54:0	3.4 ***
TAG 54:1	3.2 ***
TAG 54:2	2.5 ***
TAG 54:3	2.0 ***
TAG 54:4	2.6 ***
TAG 54:5	0.3
TAG 54:6	2.4 ***
TAG 54:7	2.3 ***
TAG 54:8	1.0 **
TAG 54:9	0.6
TAG 56:1	3.4 ***
TAG 56:2	3.3 ***
TAG 56:4	3.7 ***
TAG 56:5	2.1 ***
TAG 56:6	2.2 ***
TAG 56:7	1.1 *
TAG 58:1	3.1 ***
TAG 58:2	3.3 ***
TAG 60:2	1.7 ***
TAG 60:3	1.4 ***
