# Supplementary material for: VMP1-deficient Chlamydomonas exhibits severely aberrant cell morphology and disrupted cytokinesis
Source: BMC Plant Biol. 2014 May 6;14:121. doi: 10.1186/1471-2229-14-121 (PMC4108031; doi:10.1186/1471-2229-14-121)
Supplement: Additional file 5: Table S3 — List of qRT-PCR primers used in this work. [file 1471-2229-14-121-S5.pdf]

Ubiquitin	Cre12.g546650	TTACCTGCCTTCCGATTGCGTAGC and TTACTATGCCTGAGCACGCAGCAC
RACK1	g6364	CTTCTCGCCCATGACCAC and CCCACCAGGTTGTTCTTCAG
RCC1	Cre12.g498450	AGGTATGGAAGCCGGAGTCACT and CCGCACACCAGCTTCTTGA
RCCD1	Cre01.g017200	GTGTGAGAACACGGCAATTGC and TCCGCATCGCGTACTGATG
CYN20-2	Cre12.g544150	GACGCATGCCCCATAATGC and CCACCAGGAGGAGGTAGAAAGC
CY28	Cre12.g561000	GCTTGGCTTGCAGCTCGTAGT and TCCACCCCTGCCACTCAAC
CYN20-1	Cre06.g284900	GGACGTTGAATGCCCTAGTAAGC and CGTTCCGCTCGCACATG
PIN4	Cre16.g671900	GGGCATAGCGTGTGATGCAT and TTGCAACTATTTGCGCAGTTATGA
CYN19-2	Cre01.g002300	GAGGGCCTGACATCGGAGTT and TGCCAAATTGCTCCATGCA
CYN20-3	Cre12.g496000	CTTCCACCGCGTCATCAAG and TTGCCGGCAGTGAAGTCA
FKB15-1	Cre11.g479050	CAGCAGGGCCGAGTTTCAC and AGGTATCATAGCCGGGCGTTAC
FKB12	Cre13.g586300	CGTGTTCTTGATTTGATGGTAGTGTAAC and CCGTCCTTCGCAGTGCAA
CYN40	Cre01.g047700	CCACGTACGCACGCATGTT and CAACGGATCGCCGACTCA
CYCA1	Cre03.g207900	TTCCGTCGGCGATTAACTACTCC and TACTCCCTTGCTCACATCCTGGTG
CYCB1	Cre06.g284350	AATGGAAGAGGAGTCGCTGTTCGG and TCCAACATCACTGCAAGTCTCTGG
CYCC1	Cre03.g174476	AGAACTCGCATGCAGAAGACAGG and AAGCTCCGCGATACTTGTGC
CYCD2	Cre06.g289750	GCAACAAGAAGCCACTGTATCAGC and AGCAGTGCGTTGTGGTACTGAC
CYCD3	Cre06.g298750	ACAGCGGCGAGATGGTTATTGC and ACGCCCTCAGAATGCATGTTCC
CYCL1	Cre07.g321650	GTGTTTGACCGGATCAACAAGCG and TACTCCTTGGTCTCGGGTATCAGC
CYCM1	Cre10.g453050	AGAAGGTTCTGGCGCTTGCTAAC and GGCATAGAGCTCGTTGATTTGCG
CYCU1	Cre02.g118050	CAAGCTCACAGACGACCACTAC and GTTGATCTCCTGGACACTGACG
CDC48	Cre06.g269950	GGCGGATGTCGCCTTGA and CCCTAAGCATGCAACAATGCA
ACY1	Cre06.g271700	TTCCTTGTGTTTGCGTTGTGG and TCAAAACAGGTCCCCCAACAG
ASP1	Cre04.g226850	GCATTGACCCTACCCACTTCAC and TGAACTGCCAGTAGCCCTGG
ATG3	Cre02.g102350	GGTGCTGTTTCTCAAGTTCATCGC and ACAGACATGGTGTAGTCGTACTGG
ATG12	Cre12.g557000	CAGCAAAGCAAGGTCAAGGTCTC and CAGAGTCCGTCTTTAGCTGCTTTC
ATG6	Cre01.g020250	TTTGACAACGCGAGCGTGGATG and TGTGACCCAACAGAAGCACCTTG
ATG10	Cre12.g532300	GGATCCTCTTGAGGGAGATACAGC and TCTCGCCTACACCTTTCCTCAG
ATG4	Cre12.g510100	TCGCACTAGGCACCTACTTCTGTG and ACGAAGCCAATCGCTAGAGACG
ATG8	Cre16.g689650	CGACATTCAAGCAGGAGCATTCC and TCTGCCTTCTCGACAATGACTGG
ATG13	Cre16.g659000	TGACAAGAGAGCTGGAAGATGGC and ACGTCCTTCAAGTCCACCTCATC
UBC4	Cre02.g142000	TTCCATGCTCGACCAGAAACCG and TGTGGCACTTCCACTTCATCAGG
RPT4	Cre17.g710150	AGAGCGTGGGACAGATTATTGGC and TCAGCTTGGTCTTGTCCACCTTTG
PBE1	Cre10.g461950	CGAATGACACGAATGACCTGCAC and GGCACTTAATCCATCGCAACGC
UBP19	Cre02.g080350	AGTTGACGGCCACGCATAGAAG and GTCCGCACACAACACAATGCAC
SUMO	Cre01.g066400	TGCCTCTTTACACGCCTTGCAC and AACACCTTCTCAAGGCGGGTCTTC
Sporangin	Cre01.g049950	GTCCGTGCTTCAAATCGTGCTC and ACCACGAACTCCACATCTGTCC
